# Amelanotic Jejunal Malignant Melanoma Presenting as Overt Small-Bowel Bleeding and Mimicking a Gastrointestinal Stromal Tumor: A Case Report

**DOI:** 10.3390/life16101723

**Published:** 2026-10-15

**Authors:** Diana-Elena Floria, Ana-Maria Buzuleac, Andrei Vasile Olteanu, Răzvan Constantinescu, Dana Elena Mitrică, Gheorghe Bălan

**Affiliations:** 1 First Department of Internal Medicine, Grigore T. Popa University of Medicine and Pharmacy, 700115 Iași, Romaniabalan.gheo@me.com (G.B.); 2 Institute of Gastroenterology and Hepatology, Saint Spiridon Emergency Hospital, 700111 Iași, Romania

**Keywords:** small-bowel bleeding, jejunal melanoma, amelanotic melanoma, endoscopic ultrasound, fine-needle biopsy, enteroscopy, gastrointestinal stromal tumor

## Abstract

Background: Small-bowel bleeding accounts for a minority of gastrointestinal hemorrhage and may pose a diagnostic challenge after negative bidirectional endoscopy. Malignant melanoma involving the small intestine is usually metastatic, whereas a true primary small-bowel melanoma is exceptionally rare and remains controversial. Case Report: We report the case of a 76-year-old female patient who presented with severe symptomatic iron-deficiency anemia and recurrent overt gastrointestinal bleeding. Esophagogastroduodenoscopy and colonoscopy did not identify a bleeding source. Cross-sectional imaging demonstrated a large necrotic mass in the inter-gastro-jejunal region, initially interpreted as a possible gastrointestinal stromal tumor (GIST). Endoscopic ultrasound showed a heterogeneous lesion exceeding 100 mm and enabled fine-needle biopsy. Histopathology demonstrated a malignant epithelioid amelanotic melanoma with diffuse SOX10, S100, and Melan-A expression and a high proliferative index. Concomitant CD117 positivity represented a potential diagnostic pitfall given the initial suspicion of a GIST. Enteroscopy subsequently documented a large ulcerated jejunal lesion with friable mucosa and stigmata of recent bleeding, demonstrating luminal involvement and ascertaining it as the likely source of blood loss. Dermatologic and ophthalmologic examinations revealed no alternative primary melanoma. Conclusions: This case illustrates the complementary roles of cross-sectional imaging, EUS-guided tissue acquisition, and enteroscopy in a tumor that mimicked a GIST, while emphasizing that absence of an identifiable extraintestinal primary does not by itself prove a primary intestinal origin.

## 1. Introduction

Bleeding originating from the small bowel accounts for approximately 5–10% of gastrointestinal (GI) bleeding, which may present either overtly with melena or hematochezia, or through iron-deficiency anemia [1]. Current guidelines use the term suspected small-bowel bleeding when upper and lower endoscopic examinations do not identify a definitive source; capsule endoscopy is generally the first-line small-bowel investigation employed in these cases [2].

Malignant melanoma involving the GI tract is uncommon and most intestinal lesions represent metastases from a cutaneous or other extraintestinal primary. Primary melanoma of the small intestine is exceptionally rare; its very existence remains debated, as an occult or spontaneously regressed cutaneous primary may be impossible to rule out with certainty [3,4]. The diagnostic challenge is augmented in amelanotic tumors, which lack the characteristic pigmentation that usually suggests melanocytic differentiation.

We report the case of a large malignant amelanotic jejunal melanoma presenting with recurrent overt bleeding after negative bidirectional endoscopic assessment. The distinctive feature of this case lies in the fact that imaging findings were initially suggestive of a gastrointestinal stromal tumor (GIST). Endoscopic ultrasound (EUS)-guided fine-needle biopsy enabled tissue acquisition, and strong, diffuse SOX10, S100, and Melan-A expression supported the diagnosis of amelanotic malignant melanoma despite the potentially misleading associated CD117 positivity. Subsequent enteroscopy demonstrated ulcerated mucosal involvement with stigmata of recent bleeding. Beyond the rarity of this clinical entity, this case highlights the importance of integrating imaging, advanced endoscopy, pathology findings, and oncologic staging, while maintaining appropriate caution when assigning a primary intestinal origin.

## 2. Case Presentation

A 76-year-old female patient with a history of cardiovascular disease presented to the emergency department with melena and severe symptomatic anemia (hemoglobin 6.3 g/dL), with associated significant involuntary weight loss (8 kg over the preceding two weeks). On admission, she was conscious, cooperative, afebrile, with a mildly altered general state. Cardiopulmonary status was stable (blood pressure 140/77 mmHg, heart rate 96 bpm, SpO_2_ 97% on room air), while abdominal examination revealed an enlarged abdomen attributable to adipose tissue, without tenderness or palpable masses.

**Figure 1:**
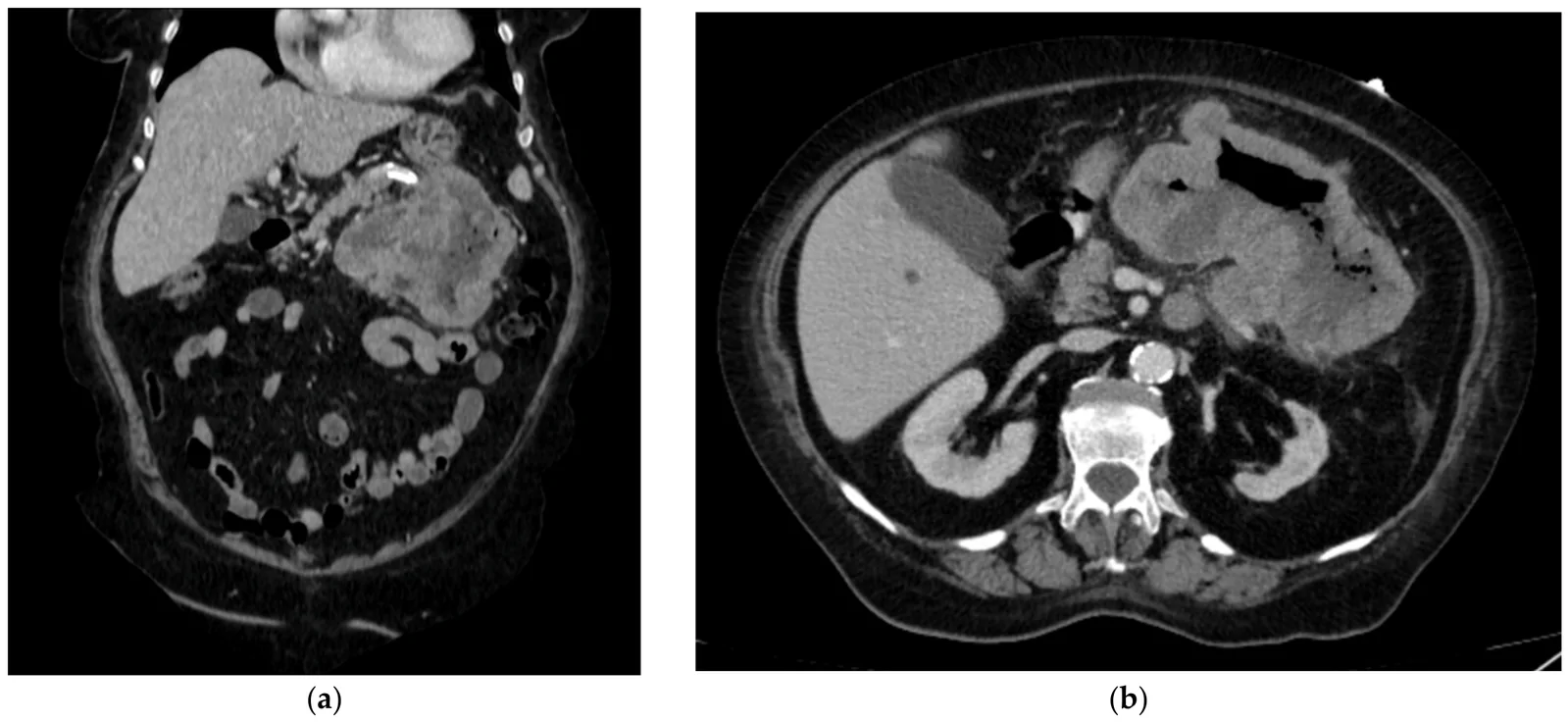
Coronal (**a**) and axial (**b**) views of contrast-enhanced computed tomography demonstrating a large expansive mass in the inter-gastro-jejunal region.

Laboratory investigations found severe hypochromic microcytic iron deficiency anemia with thrombocytosis and a nonspecific inflammatory syndrome. Initial esophagogastroduodenoscopy and colonoscopy did not identify potentially hemorrhagic lesions, suggesting possible bleeding originating from the small bowel. Abdominal ultrasonography suggested circumferential parietal thickening in the region of the transverse colon, extending on approximately 120 mm, with a maximum thickness of 40 mm, and infiltration of the adjacent adipose tissue. A subsequent contrast-enhanced abdominal and pelvic computed tomography (CT) scan revealed a large expansive mass in the inter-gastro-jejunal region of the supramesocolic compartment, measuring approximately 135 × 80 × 120 mm (Figure 1). Given the anatomic proximity of the transverse colon to the proximal jejunum, the sonographic appearance of colonic wall thickening likely reflected this same mass, with CT and subsequent EUS examinations providing the more definitive anatomic localization. The lesion contained necrotic areas and air, appeared continuous with the first jejunal loop, and lacked a clear demarcation from the posterior gastric wall, an appearance interpreted radiologically as a jejunal tumor with gastric invasion rather than a primary gastric tumor extending into the jejunum (given the mass’s epicenter and contiguity with the jejunal loop). These findings initially suggested a malignant GIST.

**Figure 2:**
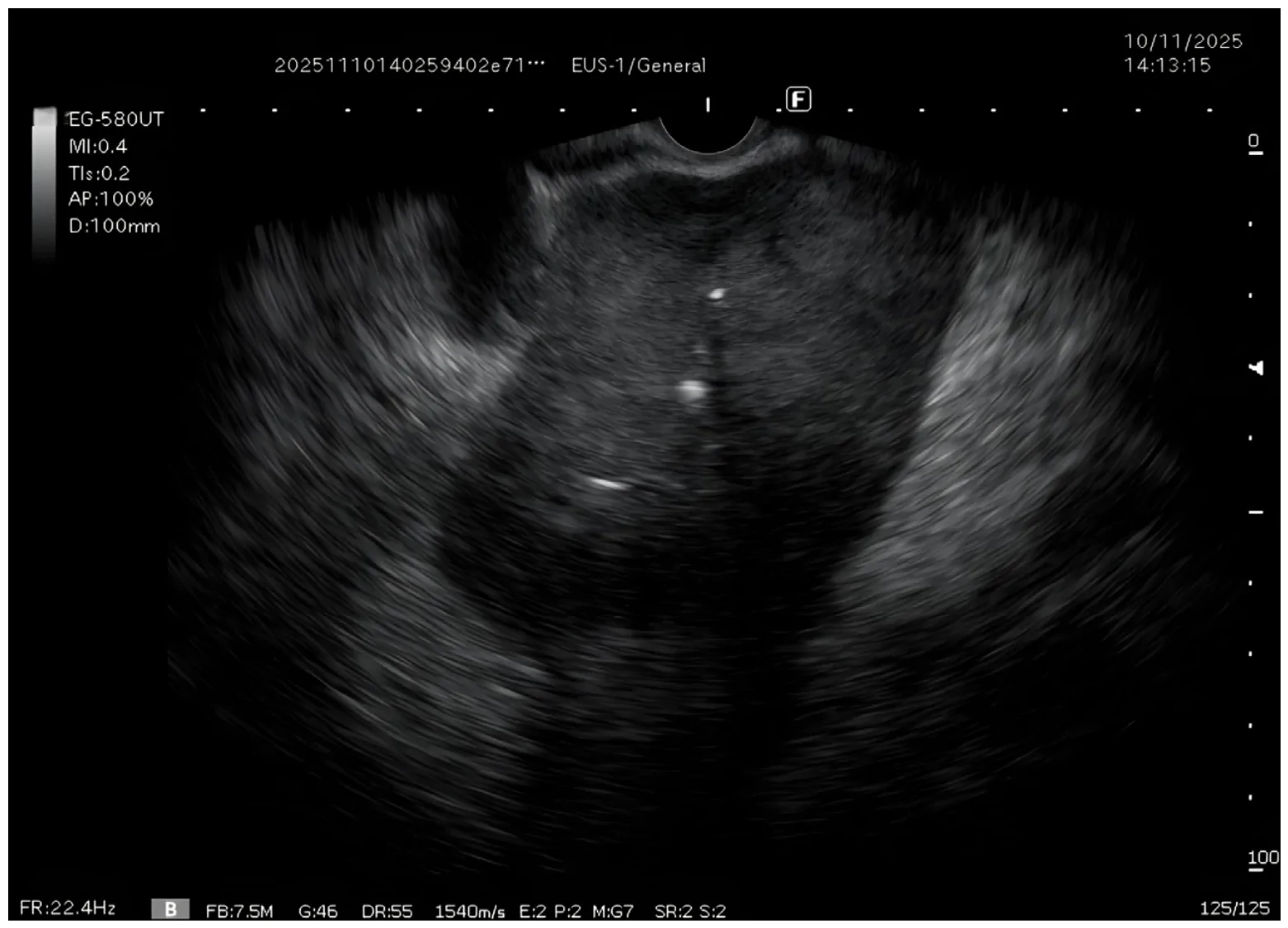
Endoscopic ultrasound examination demonstrating an irregular, heterogeneous mass with no discernible cleavage plane from the gastric wall.

Cross-sectional imaging had already identified a large mass involving the proximal jejunum, shifting the diagnostic priority from lesion detection to tissue characterization. Given the lesion’s close proximity to the stomach, EUS-guided fine-needle biopsy provided access for tissue sampling. EUS assessment further characterized the lesion as an irregular, heterogeneous mass exceeding 100 mm, with extensive necrotic components and no discernible cleavage plane from the gastric wall (Figure 2). EUS-guided fine-needle biopsy (FNB) was performed.

Histopathological examination revealed a malignant neoplasm composed of discohesive epithelioid cells with marked cytonuclear pleomorphism, multinucleation, prominent nucleoli, abundant eosinophilic cytoplasm, and extensive tumor necrosis. Immunohistochemistry demonstrated strong, diffuse expression of SOX10, S100, and Melan-A, together with CD117 positivity, whereas cytokeratin AE1/AE3 and CD34 were negative. The Ki-67 proliferation index was approximately 60%. In this morphologic and immunophenotypic context, the findings supported a diagnosis of malignant epithelioid amelanotic melanoma.

HMB-45 and additional markers to exclude other epithelioid neoplasms (including DOG1, CD45, and desmin) were not assessed because of limited availability of an extended immunohistochemical panel.

**Figure 3:**
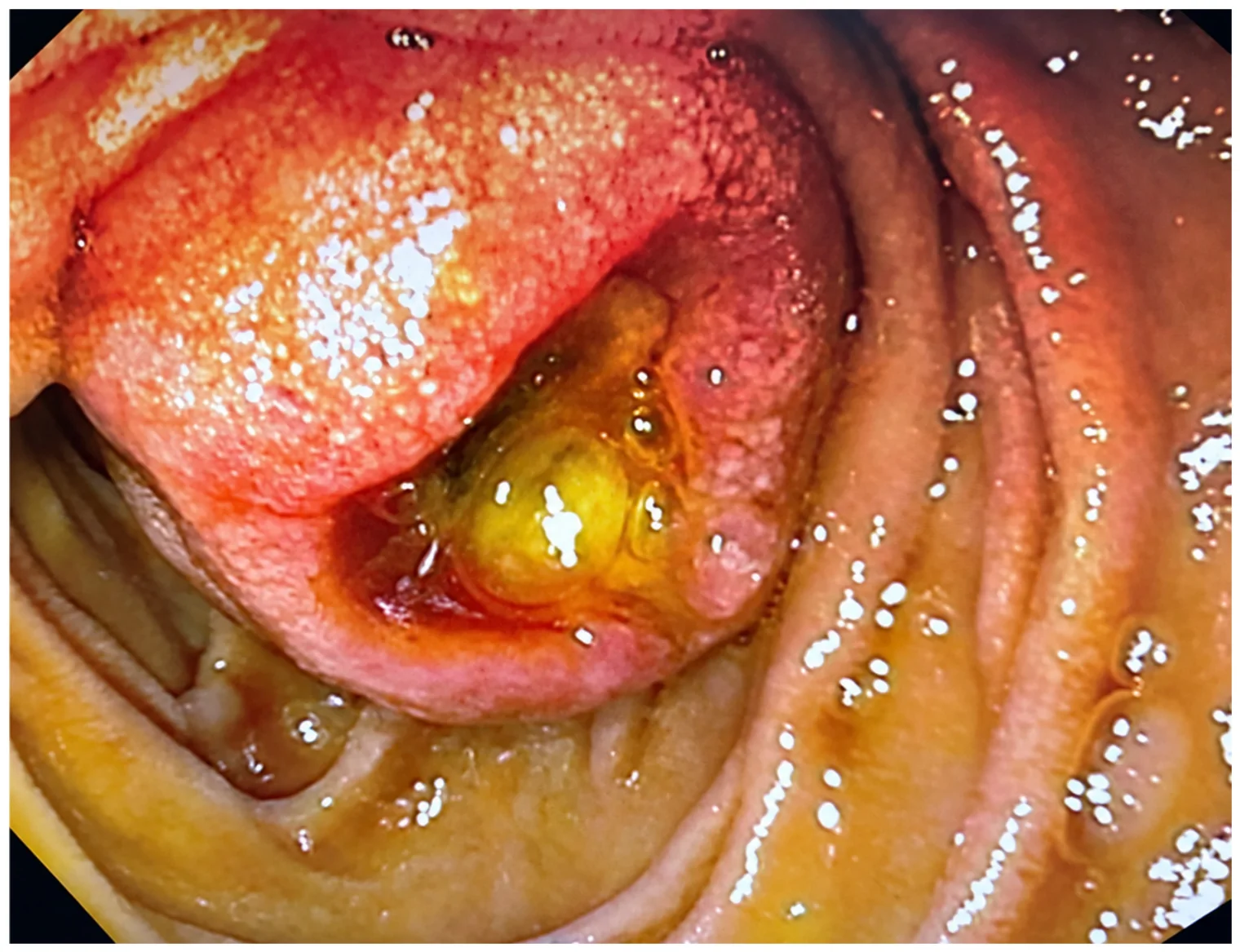
Push enteroscopy findings demonstrating a large ulcerated jejunal lesion with stigmata of recent hemorrhage.

As the anemia persisted and overt GI bleeding recurred, enteroscopy was performed following the cross-sectional and EUS-guided diagnostic work-up to directly assess small-bowel mucosal involvement and bleeding site. Enteroscopy demonstrated a large ulcerated jejunal lesion with friable mucosa and stigmata of recent hemorrhage, corresponding to the previously identified mass (Figure 3). Hence, enteroscopy confirmed the bleeding source and mucosal involvement, complementing rather than aiding in establishing the histologic diagnosis.

A comprehensive dermatologic evaluation found no suspicious cutaneous lesions, and ophthalmologic assessment showed no evidence of ocular melanoma. As no alternative extraintestinal primary melanoma was identified on the reported work-up, a primary small-bowel origin was considered.

**Figure 4:**
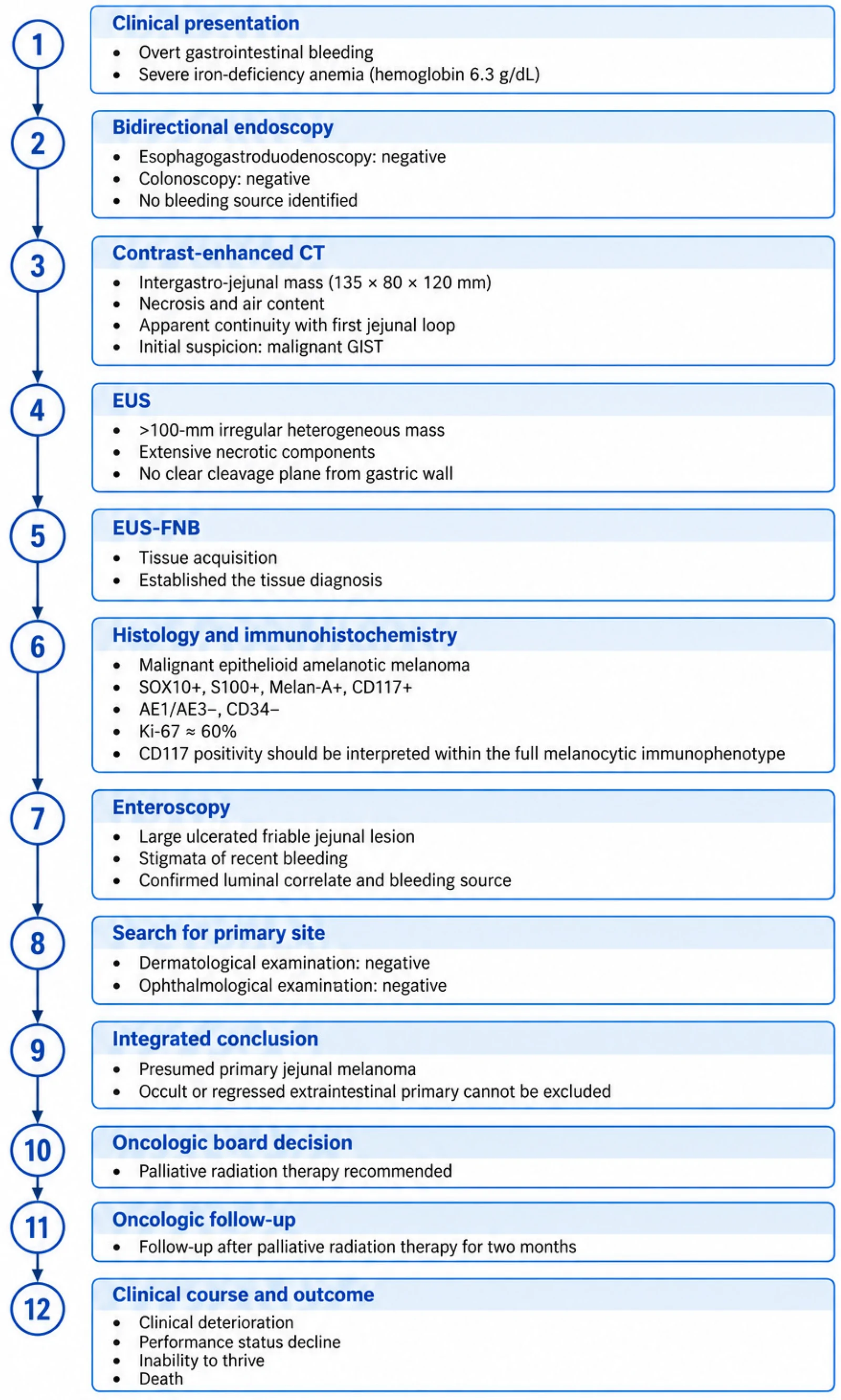
Summary of the diagnostic work-up and complementary role of each modality.

The diagnostic framework employed in this case is summarized in Figure 4.

The case was reviewed by a multidisciplinary team including general surgeons and oncologists. Full-body contrast-enhanced CT excluded distant metastatic disease. Surgical resection was not undertaken as the patient’s markedly poor performance status rendered her unfit for surgery. She was instead referred for radiotherapy, selected for its hemostatic potential given the actively bleeding tumor rather than with curative intent. She was subsequently readmitted in critical condition with hemodynamic instability and altered mental status. Her clinical course deteriorated rapidly, requiring intubation and intensive care support. She died during hospitalization approximately two months following diagnosis.

The present case does not fully satisfy the classical criteria for diagnosing a primary intestinal melanoma. Although dermatologic and ophthalmologic examinations identified no cutaneous or ocular primary, an occult or spontaneously regressed extraintestinal melanoma cannot be excluded. No surgical resection specimen was available to demonstrate an adjacent junctional or in-situ melanocytic component, and EUS-guided fine-needle biopsy could not reliably assess this feature. We therefore describe this lesion as jejunal melanoma with no identifiable extraintestinal primary, rather than as a confirmed primary intestinal melanoma.

## 3. Discussion

GI malignant melanomas may remain asymptomatic or present with nonspecific manifestations including abdominal pain, dyspepsia, unintentional weight loss, nausea, and vomiting. Advanced lesions may cause bowel obstruction, perforation, GI bleeding, iron-deficiency anemia, intussusception, or, less commonly, protein-losing enteropathy [5]. The present case illustrates two overlapping diagnostic challenges: establishing the source of overt GI bleeding and characterization of an atypical nonpigmented small-bowel tumor.

Severe iron-deficiency anemia and recurrent overt bleeding persisted despite negative esophagogastroduodenoscopy and colonoscopy, appropriately shifting attention to a small-bowel source. European Society of Gastrointestinal Endoscopy (ESGE) guidance generally supports early evaluation by capsule endoscopy in suspected small-bowel bleeding [2]; however, cross-sectional imaging in this patient had already revealed a large proximal jejunal mass. Therefore, the diagnostic pathway shifted from lesion detection to tissue characterization. EUS provided high-resolution assessment of the mass abutting the stomach and enabled tissue acquisition, ascertaining the diagnosis of malignant amelanotic melanoma. In this case, enteroscopy was complementary rather than diagnostic of the tumor itself; it demonstrated the corresponding ulcerated jejunal mucosal component and stigmata of recent bleeding, thereby linking the histologically characterized mass to the patient’s clinical presentation.

The amelanotic phenotype substantially increased diagnostic difficulty. GI melanomas may appear as polypoid, infiltrative, or ulcerated lesions, but the absence of visible melanin removes an important endoscopic and histologic clue [6]. In this patient, CT and EUS findings suggested a malignant GIST. Although CD117 positivity could have reinforced the initial suspicion of GIST, it was interpreted within the complete morphologic and immunohistochemical context, particularly the strong, diffuse expression of SOX10, S100, and Melan-A and the absence of cytokeratin AE1/AE3 and CD34 expression. Taken together, these findings supported melanocytic differentiation and a diagnosis of malignant epithelioid amelanotic melanoma. CD117 positivity was not considered independent evidence of GIST; its expression is well documented in mucosal melanomas and does not reliably predict an activating KIT mutation.

**Figure 5:**
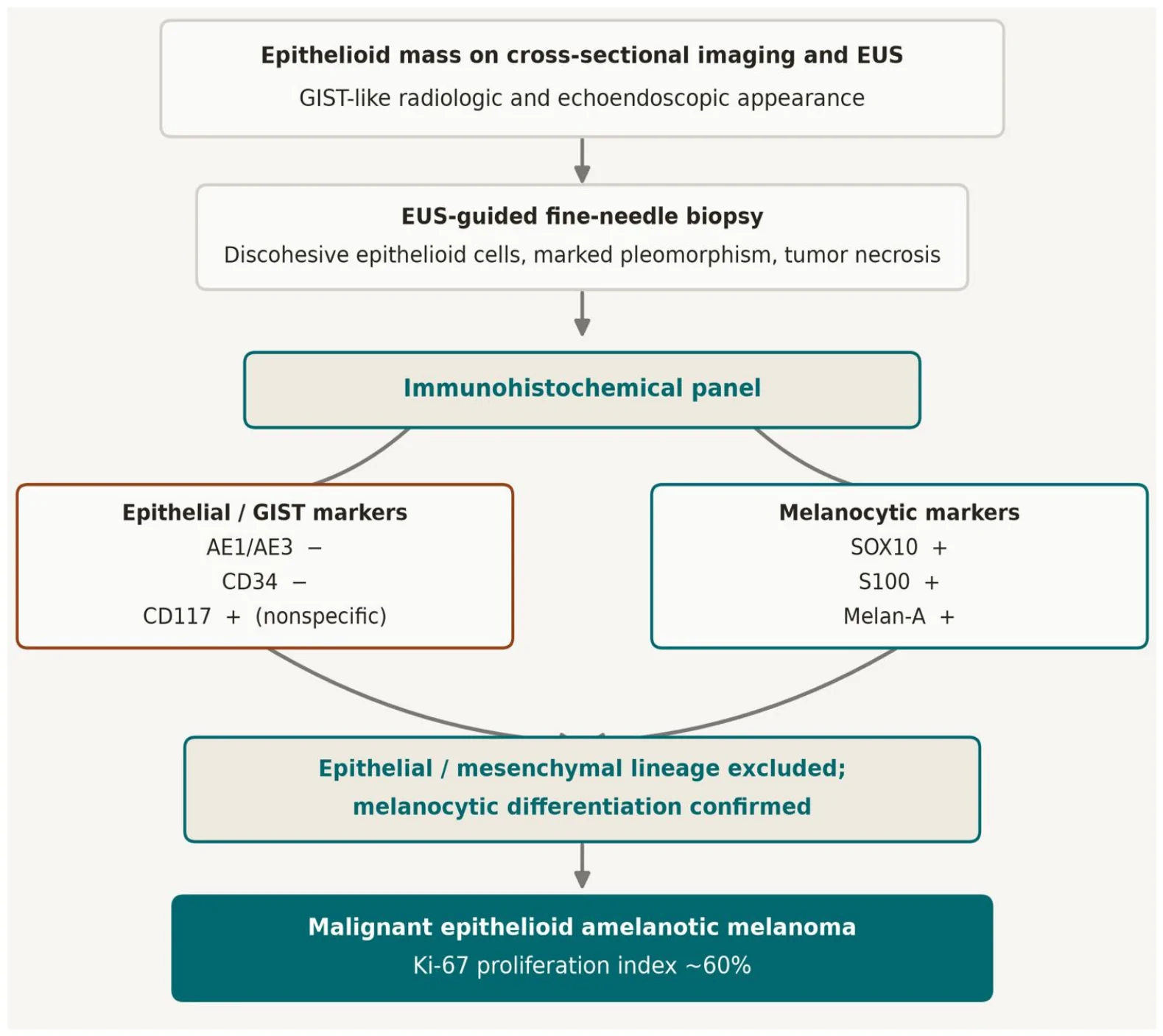
Summary of the immunohistochemical findings and diagnosis.

Molecular testing is required before KIT-directed therapy can be considered [7,8]. This combination of a GIST-like radiologic phenotype, amelanotic morphology, and CD117 expression is one of the most instructive features of the present case and is summarized in Figure 5.

Another challenging issue is whether the lesion can be classified as a true primary jejunal melanoma. The existence of primary small-bowel malignant melanomas remains controversial as melanocytes are not normally encountered in small-intestinal mucosa and because intestinal metastases from extradigestive sites are well recognized [4]. Proposed diagnostic criteria formulated by Blecker et al. emphasize the absence of a cutaneous primary and extraintestinal metastases, together with an intramucosal or junctional component adjacent to the invasive lesion [9]. Sachs et al. proposed a solitary intestinal lesion, absence of metastases beyond regional lymph nodes, and a prolonged disease-free interval as supportive features [10]. In the current case, negative dermatologic and ophthalmologic examinations and the absence of an identifiable extraintestinal primary tumor support, but do not definitively prove, an intestinal origin. EUS-FNB aided in establishing melanocytic lineage, but could not reliably evaluate the adjacent mucosa for an in-situ or junctional component. Moreover, the patient’s short survival precludes fulfillment of follow-up-based criteria. In conclusion, rather than labeling this lesion unequivocally as primary intestinal melanoma, we favor the term jejunal melanoma with no identifiable extraintestinal primary.

This distinction is not merely semantic. A recent report of ileal melanoma presenting with severe iron-deficiency anemia and active small-bowel bleeding similarly emphasized that even after complete resection, negative dermatologic and ophthalmologic examinations, whole-body metabolic staging, and prolonged disease-free follow-up, an occult regressed primary cannot be excluded absolutely [11]. In the present case, whole-body 18F-FDG PET/CT was not performed; full-body contrast-enhanced CT excluded overt distant metastatic disease. Given the patient’s markedly poor performance status, the multidisciplinary team judged curative-intent treatment, including surgery, unfeasible and adopted a palliative approach; under our national reimbursement framework, PET/CT is not a covered indication once palliative intent is established, and it was not expected to alter management. Because PET/CT can improve detection of otherwise occult metastatic melanoma and refine staging, the absence of documented whole-body metabolic staging further limits certainty regarding the site of origin [12,13].

Molecular characterization is another clinically relevant component that deserves attention in mucosal melanoma. Compared with conventional cutaneous melanoma, mucosal melanoma has a distinct genomic landscape, with lower frequencies of classic BRAF alterations and relatively greater relevance of KIT, NRAS, NF1, SF3B1, and other genomic events [8,14]. In this case, the tumor showed CD117 expression, but immunohistochemical positivity alone cannot establish an actionable KIT mutation [7]. Neither DOG1 immunostaining, nor KIT mutational testing were available; however, the intense, diffuse co-expression of SOX10, S100, and Melan-A, together with negative CD34, argued decisively against GIST despite CD117 positivity. When adequate tissue is available, management should therefore include molecular profiling for actionable alterations rather than inferring genotype from immunohistochemistry.

Management depends on disease extent, resectability, symptoms, performance status, and molecular features. Complete surgical resection is generally favored for localized, technically resectable GI melanomas and may also provide effective palliation of bleeding or obstruction; however, reported survival advantages derive largely from retrospective series and are vulnerable to selection bias [15,16]. For unresectable or metastatic mucosal malignant melanoma, immune checkpoint blockade is central to systemic treatment. Contemporary melanoma guidance supports anti-PD-1-based therapy, alone or in combination with CTLA-4 or LAG-3 blockade depending on the clinical context, rather than relegating PD-1 inhibitors to later-line therapy [17]. Radiotherapy may have a role in local control or palliation, including symptom management, but its use should be individualized within multidisciplinary care. In our case, the patient’s markedly poor performance status precluded surgical resection, and radiotherapy was selected specifically for its hemostatic potential in the setting of an actively bleeding, unresectable jejunal tumor, rather than with curative intent.

The present case has several limitations that should prompt cautious interpretation. First, the absence of a detectable cutaneous or ocular melanoma does not exclude a regressed or occult primary. Second, tissue was obtained by EUS-FNB rather than surgical resection, preventing comprehensive assessment for an adjacent in-situ or junctional melanocytic component. Third, whole-body metabolic staging and molecular profiling are not documented in the available record. Finally, the patient’s rapid clinical deterioration limited longitudinal assessment. These limitations are also what make the case educational: they illustrate why the diagnosis of primary small-bowel malignant melanoma should be expressed with caution and why multidisciplinary integration of advanced endoscopy, pathology, staging, and molecular oncology is essential.

Overall, the main value of this case lies not only in the rarity of jejunal melanoma, but also in the diagnostic work-up. Severe overt GI bleeding persisted despite negative bidirectional endoscopic evaluations, while cross-sectional imaging revealed a large GIST-like mass. EUS aided the histologic diagnosis of an amelanotic melanoma and enteroscopy demonstrated the ulcerated jejunal mucosal component responsible for digestive bleeding. This multimodal sequence may be particularly relevant when a small-bowel neoplasm has a substantial extraluminal component and cannot be adequately characterized by conventional luminal endoscopy alone.

## 4. Conclusions

Malignant amelanotic melanoma of the jejunum is an exceptionally uncommon cause of overt small-bowel bleeding and may closely mimic a GIST on imaging. This case illustrates the value of multimodal assessment when a small-bowel mass is identified: cross-sectional imaging characterized the lesion, EUS-guided biopsy established the diagnosis of amelanotic melanoma, and enteroscopy documented jejunal mucosal involvement and the bleeding source. Primary intestinal origin should be assigned cautiously, as the absence of an identifiable extraintestinal primary does not establish intestinal origin. Given the unmet diagnostic criteria, “jejunal melanoma with no identifiable extraintestinal primary” more accurately reflects the residual uncertainty.

## Data Availability

The original contributions presented in this study are included in the article. Further inquiries can be directed to the corresponding author(s).

## Abbreviations

The following abbreviations are used in this manuscript:

GI	Gastrointestinal
GIST	Gastrointestinal Stromal Tumor
EUS	Endoscopic Ultrasound
CT	Computed Tomography
FNB	Fine Needle Biopsy
